# “我爱实验室”科研训练：一种阳离子型共价有机框架气凝胶用于有机染料去除

**DOI:** 10.3724/SP.J.1123.2025.10018

**Published:** 2026-07-08

**Authors:** Yunmeng LI, Hongyi LI, Dongxiao CAO, Anna TANG, Deming KONG

**Affiliations:** 南开大学化学学院，分析科学研究中心，天津市生物传感与分子识别重点实验室，化学国家级实验教学示范中心，天津 300071; Research Center for Analytical Sciences，Tianjin Key Laboratory of Biosensing and Molecular Recognition，National Demonstration Center for Experimental Chemistry Education，College of Chemistry，Nankai University，Tianjin 300071，China

**Keywords:** 离子型共价有机框架, 甲基橙, 吸附去除, 教学实验, ionic covalent organic frameworks, methyl orange, adsorption removal, teaching experiment

## Abstract

甲基橙（MO）是一种典型的水溶性偶氮类有机染料。在基础实验中常被用作酸碱指示剂，在工业生产中是一种常见的有机染料，对水体具有强污染性。如果不慎食用偶氮染料，偶氮键被体内偶氮还原酶裂解，生成的芳香胺毒性大且有致癌风险。离子型共价有机框架（ICOFs）是共价有机框架（COFs）材料的框架和孔道上带有电荷的新型材料。ICOFs在水溶液中展现出良好的吸附性能，其高比表面积和丰富的多孔通道有利于污染物快速扩散到内层结构中，能有效吸附污染物。将具有规则的垂直大孔的绿色物质茄子作为基质，通过原位生长法制备出一种对MO高效吸附的阳离子型共价有机框架茄子复合气凝胶（EP@PDA-TGDha COF），并将其应用于有机染料污水处理中。将该实验开发为本科生“我爱实验室”科研训练实验，有助于培养和提升学生在样品前处理材料合成、材料结构和性质表征及实际样品分析方面的实验技能和独立分析问题、解决问题的能力。学生通过线下接触优秀课题组，感受科研氛围，了解学术前沿，跨出学术道路关键的第一步。通过“我爱实验室”科研训练，学生的专业知识储备、实验技能及科研思维能力均得到大幅提高。同时将思政元素有机融入科研训练中，培养学生的环境保护意识。

## Abstract (en)

Methyl orange （MO） is a typical water-soluble azo organic dye. It is commonly used as an acid-base indicator in basic experiments and as a common organic dye in industrial production. It has strong polluting effects on water bodies. If azo dyes are accidentally ingested， the azo bond is cleaved by azoreductase， generating highly toxic aromatic amines that pose potential carcinogenic risks. Ionic covalent organic frameworks （ICOFs） are novel materials derived from covalent organic frameworks， featuring charged structures on their frameworks and pores. ICOFs exhibit excellent adsorption performance in aqueous solutions. Their high surface area and abundant porous channels facilitate rapid diffusion of pollutants into the inner structure， enabling effective pollutant adsorption. Eggplant is a sustainable biomass material with regular vertical macropores， which can be used as a substrate. This experiment employed an in-situ solvothermal method to prepare a highly efficient adsorbent monolithic material of eggplant composite aerogel （EP@PDA-TGDha COF）. It is applied to organic dye wastewater treatment. Developing this experiment as an undergraduate teaching module of “I love the laboratory” helps cultivate and enhance students’ abilities in sample pretreatment， material synthesis， structural and property characterization， and practical sample analysis. Students enter the research group offline， experience the academic atmosphere， keep abreast of cutting-edge research， taking the crucial first step in their academic journey. Through the “I Love the Lab” research training program， students significantly enhance their professional knowledge， experimental skills， and research-oriented thinking abilities. It also fosters students’ environmental protection awareness and enables them to apply their knowledge and skills to environmental conservation.

随着染料工业的蓬勃发展，如何处理含有有机染料残留物的废水成为人们日益关注的问题。偶氮染料，顾名思义为化学结构中含有偶氮键的染料，通常在偶氮键两端连有芳基，可以根据所含偶氮键数量分为单偶氮类、双偶氮类和多偶氮类。如果不慎食用偶氮染料，偶氮键被体内偶氮还原酶裂解，生成的芳香胺毒性大且有致癌风险^［1-3］^。甲基橙（MO）在基础实验中常被用作酸碱指示剂，在工业生产中是一种常见的有机染料，对水体具有较强的污染性并且难以降解。

共价有机框架（COFs）是一类由轻质元素（C、H、O、N、B等）通过较强共价键（如硼氧键、碳碳双键、碳氮双键等）连接，具有二维或三维结构的结晶性多孔高分子材料^［4-8］^。COFs密度低且易修饰，具有良好的化学稳定性和热稳定性。离子型共价有机框架（ICOFs）是在COFs框架和孔道上带有电荷的新型材料^［9-13］^。ICOFs在水溶液中展现出良好的吸附性能，其高表面积和丰富的多孔通道有利于污染物快速扩散到内层结构中，能有效吸附污染物。在污水处理中，可以通过调控COFs孔隙结构和表面功能基团，实现对不同类型污染物的选择性吸附和去除。

采用木质素、软木、玉米秸秆等生物质材料作为COFs的支撑基质已有相关报道^［14-16］^。相比于这些材料，茄子绿色可再生，具有独特的垂直孔道结构，使用简单的冷冻干燥便可制成低密度的气凝胶。我们选取茄子^［17，18］^作为支撑基质，通过溶剂热法制备了一种对甲基橙高效吸附的整体材料茄子复合气凝胶（EP@PDA-TGDha COF），并通过多种表征手段验证了其稳定性和结晶性。本材料在最佳pH条件下对MO的吸附容量高，循环利用性能好，吸附完成后，可以直接用镊子夹出，省去了离心等烦琐步骤。与粉末材料相比不易泄漏到环境中，避免造成二次污染，有望应用于有机染料污水处理中。

“我爱实验室”活动由南开大学化学学院学生科技创新协会承办，面向化学学院全体大一新生开展。该活动旨在帮助我院本科大一新生培养科研兴趣、树立科研目标，通过线下接触优秀课题组，感受科研氛围，了解学术前沿，跨出学术道路关键的第一步，甚至锁定未来心仪的导师及课题组，是学院本科生科研能力提升体系中的重要环节之一。将该实验开发为本科生“我爱实验室”科研训练实验，有助于培养和提升学生将专业理论知识应用于实际样品分析的能力。通过对样品前处理材料的合成、材料的结构和性质表征及实际样品分析，使学生掌握样品前处理材料的合成方法；熟悉多种分析仪器的性能及使用；掌握分离富集实验的设计过程；培养和提升学生的科研兴趣和科研能力，同时培养学生“学以致用”、保护生态环境的思想意识。

## 1 实验部分

### 1.1 仪器、试剂与材料

Vortex-Genie 2涡旋振荡器（美国Thermo Fisher公司）；TENSOR Ⅱ红外光谱仪（IR，德国Bruker公司）；Rigaku SmartLab X射线粉末衍射光谱仪（PXRD，日本理学公司）；Apreo S LoVac扫描电子显微镜（SEM）、Tecnai G2 F20透射电子显微镜（TEM，捷克FEI公司）；Zetasizer Nano ZS90纳米粒度电位仪（英国Malvern公司）；CTFD-10冷冻干燥机（永合创信电子科技有限公司）；TG209 DSC204 DMA242热分析系统（德国NETZSCH公司）；ASAP 2460全自动比表面及孔隙度分析仪（美国Micromeritics公司）；Scientific LSA100表面张力测试仪（德国Lauda公司）；UV-Cary 60紫外可见分光光度计（美国Agilent公司）；AVANCE NEO 400 MHz固体超导核磁共振谱仪（NMR，瑞士Bruker公司）。

三氨基胍盐酸盐（TGCl）、氢氧化钠（NaOH）、碳酸钠（Na_2_CO_3_）均购于上海麦克林生化科技有限公司；2，5-二羟基对苯二甲醛（2，5-Dha）购于上海毕得医药科技有限公司；盐酸多巴胺（PDA）、三羟甲基氨基甲烷盐酸盐（Tris-HCl）均购于上海阿拉丁生化科技有限公司；甲基橙、亚甲基蓝（MB）均购于北京百灵威科技有限公司；盐酸（HCl）、硝酸（HNO_3_）、甲醇（CH_3_OH）、四氢呋喃（THF）均购于天津康科德科技有限公司。所有化学试剂均为分析纯及以上纯度，无需进一步纯化处理。

### 1.2 实验条件

#### 1.2.1 EP@PDA-TGDha COF气凝胶的制备

对购买的新鲜茄子进行去皮处理，并切成1 cm×1 cm×1 cm的立方体块状。配制10 mmol/L的Tris-HCl缓冲溶液50 mL，调节pH至8.5后加入10 mg盐酸多巴胺并使其充分溶解。将上述盐酸多巴胺溶液与新鲜立方体茄块混合，然后在室温下搅拌8 h，使盐酸多巴胺自聚于茄子表面。自聚完成后，用纯净水冲洗立方体茄块3~5次，将其置于‒80 ℃冷冻3 h。随后将立方体茄块冷冻干燥20 h，即可得到深黑色的氨基化茄子气凝胶（EP@PDA）。

准确量取6.8 mL四氢呋喃和1.2 mL纯净水，将二者混合均匀后，先后加入56.2 mg三氨基胍盐酸盐和99.7 mg 2，5-二羟基对苯二甲醛，超声处理25 min直至完全溶解。将上述溶液放入涡旋振荡器混合3 min，直至彻底混合变为棕黄色。加入4块EP@PDA气凝胶，继续涡旋20 min，直至混合物变为橙黄色。随后将混合物转移至聚四氟乙烯内胆，密封至不锈钢反应釜内。将反应体系的温度提高到120 ℃，在此温度下连续反应72 h（使用高压反应釜，额定耐压≥3 MPa）。反应结束后自然冷却至室温，使用四氢呋喃和甲醇依次洗涤立方体茄块，直至上清液变得澄清。然后用纯净水冲洗立方体茄块3~5次，将其置于‒80 ℃冷冻3 h。随后将立方体茄块冷冻干燥20 h，即可得到橙棕色的茄子复合气凝胶（EP@PDA-TGDha COF）。图1为TGDha COF的合成反应。

**图1:**
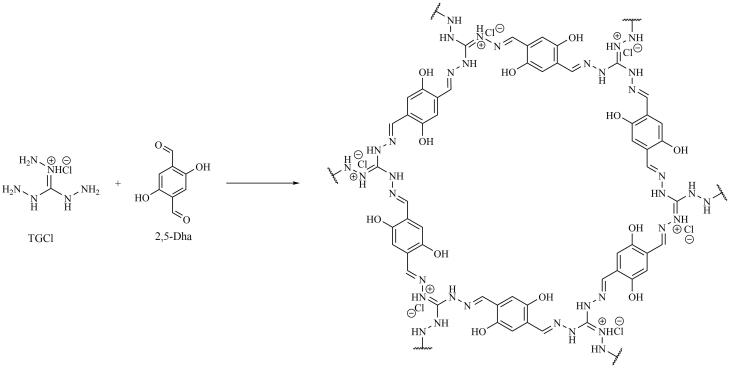
TGDha COF材料的合成反应

#### 1.2.2 吸附热力学实验

在pH=6条件下，分别配制100、200、300、400、500、600、1 000 mg/L的MO溶液。准确称量3 mg EP@PDA-TGDha COF，转移至5 mL离心管中。随后分别在pH=6条件下加入3 mL不同浓度的MO溶液，设置涡旋振荡器转速为1 500 r/min，将离心管置于其中涡旋5 h至吸附平衡。吸附结束后过膜，利用紫外分光光度计测量上清液的吸光度。吸附后的数据，利用Langmuir（式（1））和Freundlich（式（2））吸附等温模型处理，研究室温下EP@PDA-TGDha COF的热力学性质。


$$\frac{c_{e}}{q_{e}}=\frac{c_{e}}{q_{max}}+\frac{1}{bq_{max}}$$ （1）

$$ln\;q_{e}=ln\;K_{F}+\frac{1}{n}ln\;c_{e}$$ （2）

其中，*c*_e_（mol/L）为吸附平衡后MO的浓度，*q*_e_（mg/g）为吸附平衡后EP@PDA-TGDha COF对MO的吸附量，*q*_max_（mg/g）为最大吸附量，*b*（L/mg）为Langmuir常数，*K*_F_和*n*代表Freundlich常数，分别表示吸附量和吸附强度。

#### 1.2.3 吸附动力学实验

在pH=6条件下，配制10 mg/L的MO溶液。准确称量3 mg EP@PDA-TGDha COF，转移至5 mL离心管中。随后在pH=6条件下加入3 mL 10 mg/L的MO溶液，设置涡旋振荡器转速为1 500 r/min，将离心管置于其中涡旋，涡旋时间分别为0.5、1、2、4、6、8、10 min。吸附结束后过膜，利用紫外分光光度计测量上清液的吸光度。吸附后的数据，利用准一级动力学模型（式（3））和准二级动力学模型（式（4））处理，研究EP@PDA-TGDha COF的动力学性质。


$$ln\;(q_{e}-q_{t})=ln\;q_{e}-k_{1}t$$ （3）

$$\frac{t}{q_{t}}=\frac{1}{k_{2}}\frac{1}{{q_{e}}^{2}}+\frac{t}{q_{e}}$$ （4）

其中，*q_t_* （mg/g）为吸附时间 t （min）时EP@PDA-TGDha COF对甲基橙的吸附量，*k*_1_（min^‒1^）为准一级动力学常数；*k*_2_为准二级动力学常数（g/（mg·min））。

#### 1.2.4 实际水样吸附实验

实际样品选用自来水、新开湖、小引河的水样以及在上述实际水样中加标10 mg/L甲基橙溶液的加标样品，用1 mol/L盐酸和1 mol/L氢氧化钠溶液调至pH=6。准确称量3 mg EP@PDA-TGDha COF，转移至5 mL离心管中，随后分别加入3 mL样品溶液。将离心管置于涡旋振荡器中，设置转速为1 500 r/min，涡旋10 min。吸附结束后过膜，利用紫外分光光度计测量上清液的吸光度。MO的去除率（*R*，%）可由式（5）计算得出：


$$R=\frac{c_{0}-c_{e}}{c_{0}}\times 100\%$$ （5）

其中，*c*_0_（mol/L）为吸附前MO的初始浓度，*c*_e_（mol/L）为吸附平衡后MO的浓度。

## 2 结果与讨论

### 2.1 EP@PDA-TGDha COF材料的表征

如图2a所示，TGDha COF材料和EP@PDA-TGDha COF都在1 612 cm^‒1^处有明显的C=N的特征吸收峰，而C=O的吸收峰消失，证明了席夫碱反应的成功进行。对材料进行^13^C核磁共振分析。如图2b所示，*δ* 121和114处的信号峰来自两个O-H内侧的碳原子，*δ* 150处的信号峰来自苯环碳原子，在*δ* 154处的信号对应TGCl的中心碳原子。核磁共振谱图进一步证明了碳键之间的连接方式。

**图2:**
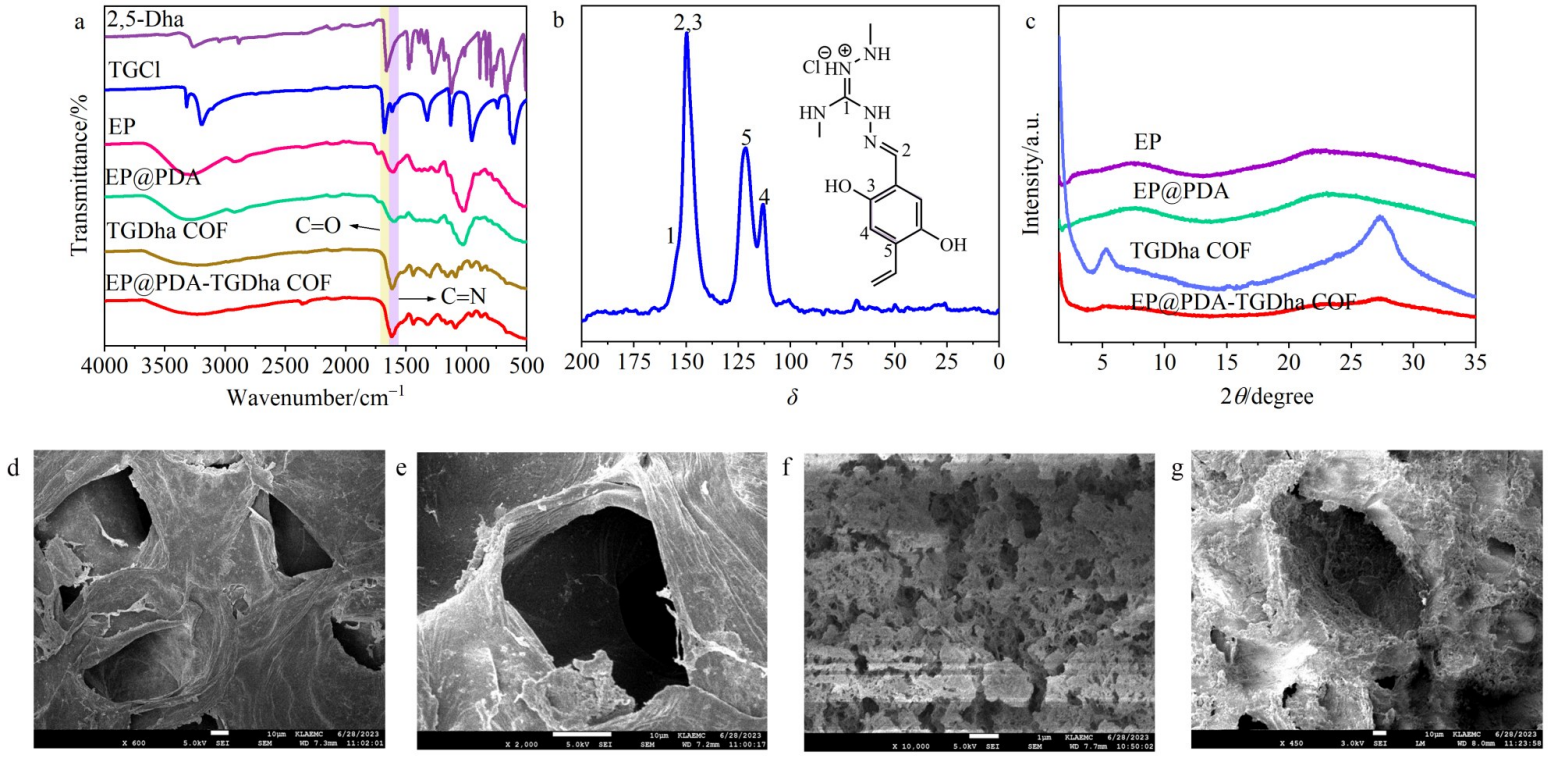
**（**a）2，5-Dha、TGCl、EP、EP@PDA、TGDha COF和EP@PDA-TGDha COF的红外光谱图；（b）TGDha COF的^13^C核磁共振谱图；（c）EP、EP@PDA、TGDha COF和EP@PDA-TGDha COF的PXRD图；（d，e）EP、（f）TGDha COF粉末和（g）EP@PDA-TGDha COF的扫描电镜图

为探究EP@PDA-TGDha COF的结晶度，对材料进行X射线粉末衍射分析，如图2c所示。从图中可以明显看出，EP和EP@PDA不结晶，EP@PDA-TGDha COF有两个微弱的衍射峰，与TGDha COF材料的衍射峰相对应，代表了同样的晶面和晶体结构。衍射峰不明显主要是因为EP@PDA-TGDha COF中EP占比较大。

从扫描电镜结果可以看出，EP表面光滑有较大孔隙（图2d和2e），TGDha COF粉末形貌为丝状网状相互交错（图2f）。用TGDha COF进行修饰后，EP表面不再光滑，出现了与TGDha COF形貌相符的丝状物质（图2g），进一步证明了TGDha COF的成功生长。

为探究EP@PDA-TGDha COF的孔隙结构，对材料进行了BET表征。如图3a所示，氮气吸脱附实验证明了EP@PDA-TGDha COF表现为Ⅰ型和Ⅳ型吸附等温线的组合。由图3b可知，EP@PDA-TGDha COF的孔径在1.5 nm左右。对材料进行热重分析，如图3c所示，在250 ℃时，失重大约为10%，证明EP@PDA-TGDha COF具有一定的热稳定性。为探究EP@PDA-TGDha COF表面所带电性，对材料进行Zeta电位分析。如图3d所示，pH值为2~7时，EP@PDA-TGDha COF的Zeta电位为正值；pH值为8~10时，EP@PDA-TGDha COF的Zeta电位为负值。说明在酸性与中性条件下材料带正电，可以利用静电相互作用吸附MO。

**图3:**
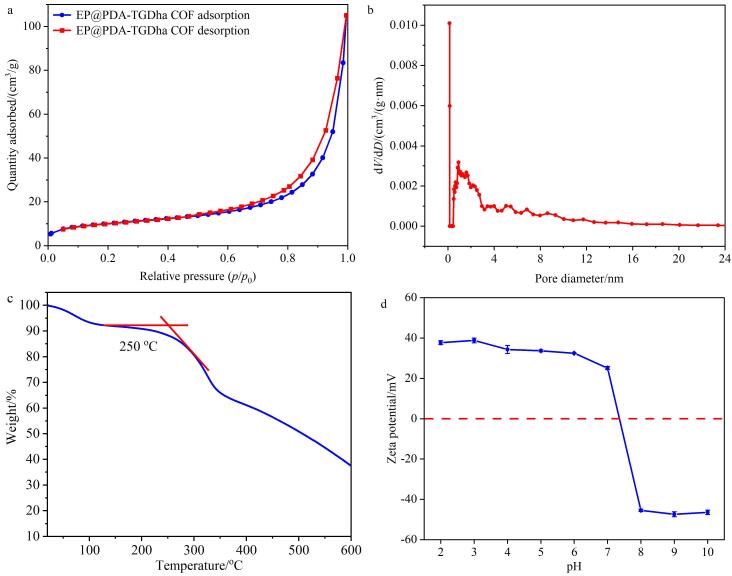
EP@PDA-TGDha COF的（a）N_2_吸附-脱附等温曲线、（b）孔径分布、（c）热重曲线和（d）Zeta电位图

在动态水接触角测试中（图4），可以明显看出EP和EP@PDA疏水性较强，而水滴在接触到TGDha COF和EP@PDA-TGDha COF时，50 ms就已被全部吸收。这说明TGDha COF修饰改善了材料的亲水性，更有利于接触水中的目标物，从而更好地发挥吸附作用。

**图 4:**
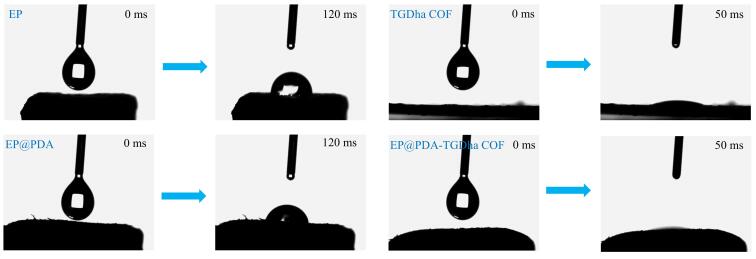
EP、EP@PDA、TGDha COF和EP@PDA-TGDha COF在室温下的水接触角

为探究EP@PDA-TGDha COF的酸碱稳定性，分别对其用3 mol/L盐酸、3 mol/L氢氧化钠溶液和*N，N*-二甲基甲酰胺（DMF）浸泡3天，随后进行红外光谱分析和X射线粉末衍射分析。如图5所示，经过酸碱浸泡处理后的EP@PDA-TGDha COF在C=N的红外特征吸收峰和X射线衍射峰与未处理过的EP@PDA-TGDha COF保持一致，证明了EP@PDA-TGDha COF具有好的酸碱稳定性。

**图5:**
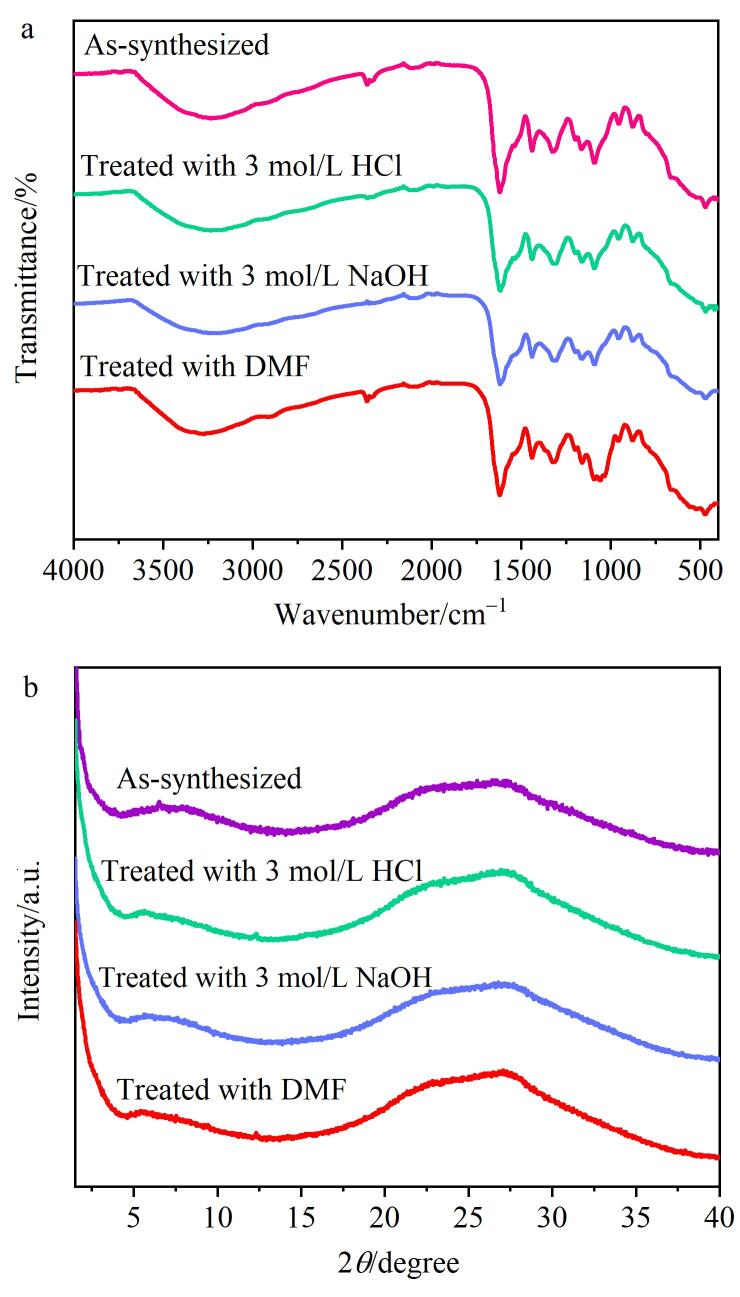
未处理过和分别用HCl、NaOH溶液和DMF浸泡3天的EP@PDA-TGDha COF的（a）红外光谱图和（b）PXRD图

### 2.2 吸附实验pH值的优化

如图6所示，EP@PDA-TGDha COF在pH值为2~7的条件下对甲基橙的去除率均在90%以上，而在pH值为8~10的条件下对甲基橙的去除率则不足10%。实验结果与Zeta电位分析相对应。pH值为2~7时，EP@PDA-TGDha COF带正电，可与甲基橙发生静电相互作用，从而更高效地吸附甲基橙。综合考虑，选择pH=6为后续吸附条件。

**图6:**
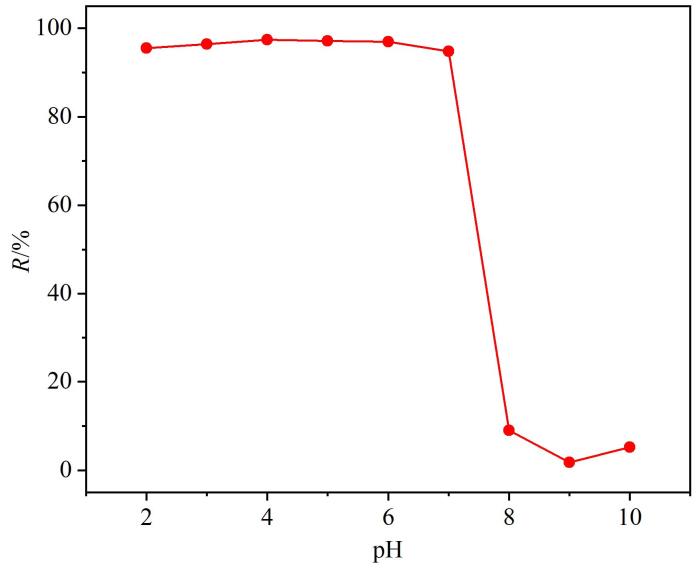
不同pH条件下EP@PDA-TGDha COF对甲基橙的去除率

### 2.3 吸附热力学

如图7所示，Langmuir模型拟合的*R* ^2^=0.992 8，Freundlich模型拟合的*R* ^2^=0.973 3，可知吸附过程符合Langmuir模型。这说明吸附剂表面均匀，对MO的吸附主要为单分子层吸附。EP@PDA-TGDha COF对MO染料展现出良好的吸附性能，根据Langmuir模型计算出的最大吸附容量为719.42 mg/g。

**图7:**
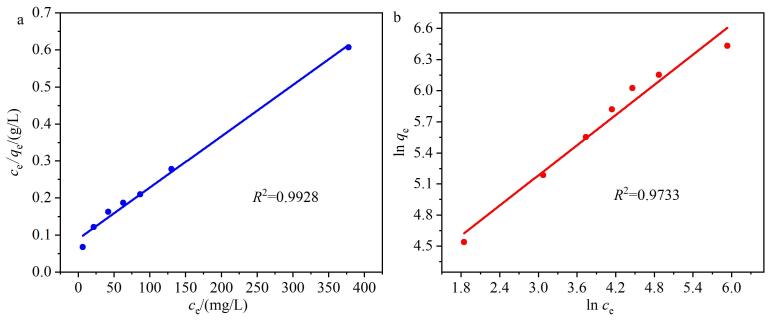
（a）Langmuir和（b）Freundlich等温吸附模型

### 2.4 吸附动力学

拟合结果表明，EP@PDA-TGDha COF对MO染料的动力学吸附行为更符合准二级动力学模型（图8）。这说明吸附剂对MO的吸附过程主要受化学作用控制，且涉及吸附剂与MO之间电子的共用和转移。

**图8:**
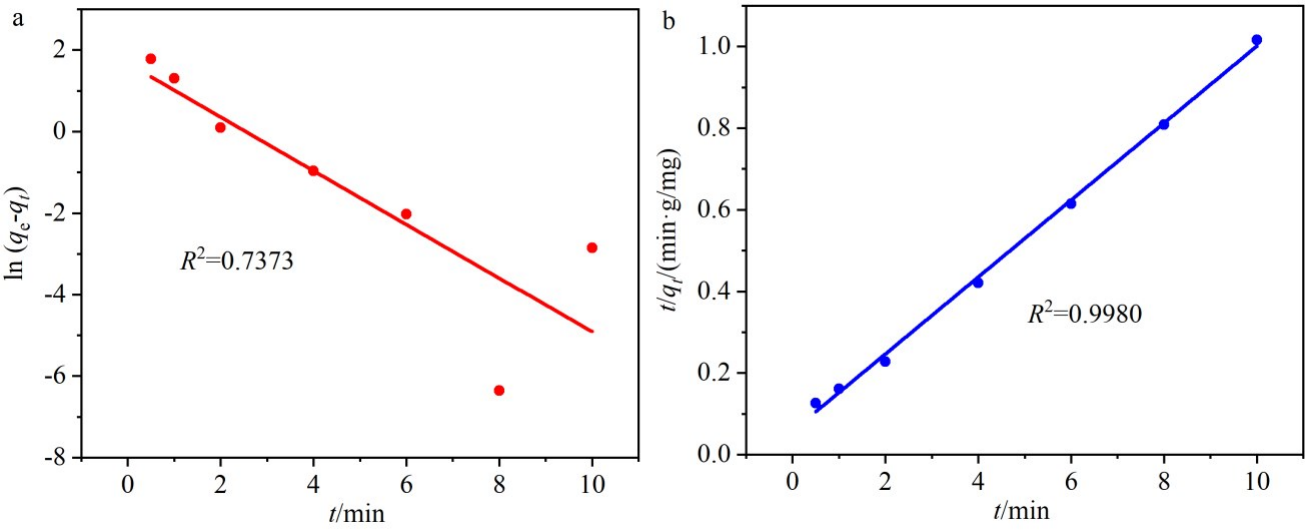
EP@PDA-TGDha COF对MO吸附过程的（a）准一级动力学模型和（b）准二级动力学模型

### 2.5 吸附选择性

为验证EP@PDA-TGDha COF对阴离子染料MO的选择性，用常见的阳离子染料MB作为干扰进行吸附实验。如图9a所示，橙色的MO和蓝色的MB混合后呈紫色，经过吸附处理后又变回蓝色，吸附效果明显。从紫外可见吸收光谱（图9b）可以明显地看到MO的吸收峰在吸附实验后消失，而MB的吸光度值基本不变。这证明了EP@PDA-TGDha COF通过静电相互作用实现了对MO的选择性吸附。

**图9:**
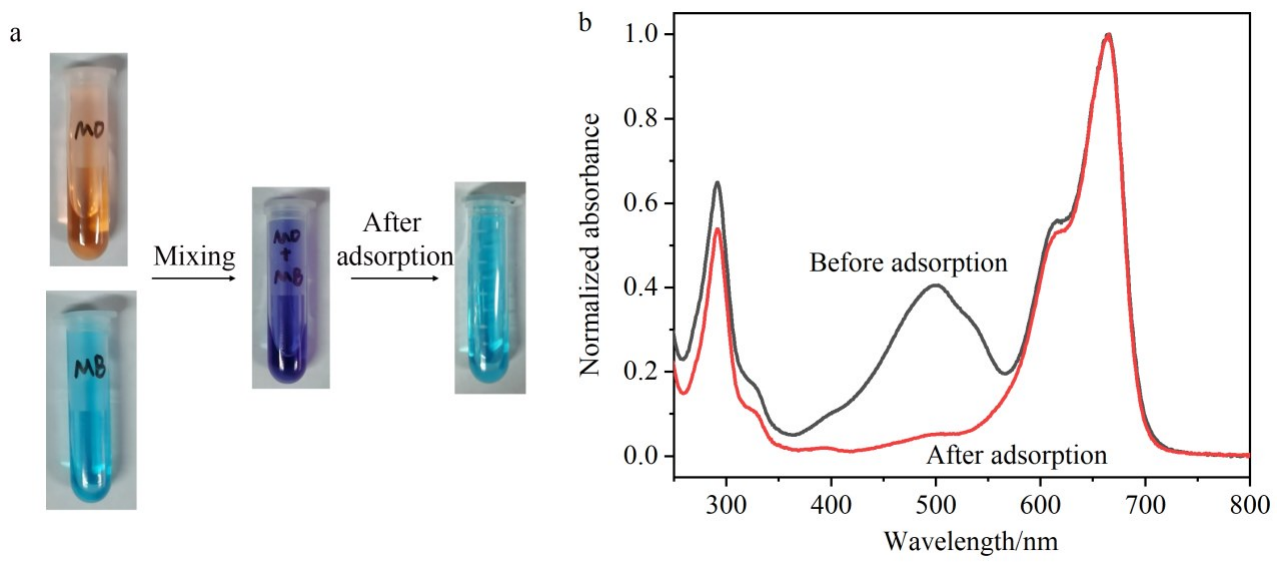
（a）EP@PDA-TGDha COF对MO与MB混合溶液的选择性吸附及其（b）吸附前后的紫外可见吸收光谱图

### 2.6 实际水样应用

为探究EP@PDA-TGDha COF在实际环境样品水样中的应用前景，分别取自来水、新开湖和小引河的实际水样进行吸附实验。在自来水、新开湖和小引河的实际水样中均未检测到MO。在自来水和小引河的实际水样中加标后得到模拟真实污水样品，对其进行测定，MO的去除率可达96%以上，新开湖的加标样品中，MO的去除率略低，但仍在92%以上（如图10所示）。

**图10:**
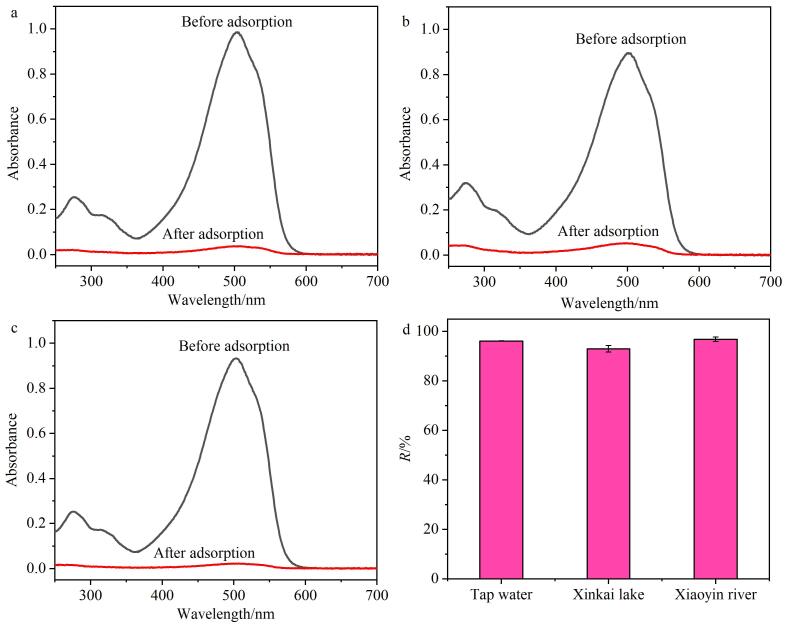
（a）自来水、（b）新开湖和（c）小引河实际加标水样吸附前后的紫外可见吸收光谱图；（d）不同实际加标水样中EP@PDA-TGDha COF对MO的去除率（*n*=3）

## 3 实验的组织实施及教学反思

### 3.1 组织实验室参观学习

首先组织学生参观科研实验室，向学生介绍课题组的科研方向，介绍在样品前处理分离富集实验中，常用到的材料合成方法、仪器设备、实验室内的操作规范与安全常识。鼓励本科生与实验室的硕士、博士沟通交流，了解科研生活。推荐给学生一些与课题相关的文献、资料。

### 3.2 查阅文献，熟悉背景知识

学生通过文献调研，深刻认识到废水中有机染料去除的重要意义；了解目标物甲基橙的性质；采用样品前处理方法的必要性及常用的样品前处理方法；阳离子型COFs材料的合成方法及性质；阳离子型COFs材料与目标物的可能作用机理；吸附实验的设计过程；实际废水样品分析。

### 3.3 明确实验目的

学生与指导教师开展课题讨论，明确实验目的。（1）开发一种样品前处理材料茄子复合气凝胶EP@PDA-TGDha COF；（2）将该材料用于实际环境水样中有机污染物甲基橙的去除；（3）掌握样品前处理材料EP@PDA-TGDha COF的合成方法、各种性质表征手段及吸附机理；（4）培养保护生态环境的思想意识。

### 3.4 科研训练实验的组织

“我爱实验室”科研训练实验面向南开大学化学学院大一本科生开展，是学院本科生科研能力提升体系中重要环节之一。学生在学习了基础化学理论、分析化学专业课的前提下，可以根据自身的兴趣，选择合适的课题组进行科研训练。学生按组制订科研训练计划和人员安排，利用课余时间进入实验室，在教师和学长的指导下，开展科研实验。整个科研训练时间安排一个学期（17周）完成。具体安排如下：1~2周学生到实验室参观学习，熟悉实验室环境，特别注意实验室内的操作规范与安全常识；3~4周调研文献、资料，了解与课题相关的背景知识；5~8周学生在教师和学长的指导下开展吸附材料的合成实验；9~10周完成吸附材料的性质表征，并基于表征结果进一步优化合成条件；11~14周开展吸附去除实验的考察；15~16周应用于实际样品分析，同时继续完善前期的实验工作。17周整理、总结实验结果，提交实验报告。

在科研训练过程中，学生要注意平衡课堂学习和课下科研训练的时间安排，将理论学习与科研实践有机结合，相辅相成。在实验过程中，教师发挥引导作用，指导学生查阅文献资料，多实践尝试，充分发挥学生学习主动性，培养学生独立思考、分析和解决问题的能力。

### 3.5 实验教学中存在的不足及解决措施

在最初的实验过程中，我们发现学生的专业知识储备、实验技能及科研思维能力欠缺。

针对学生专业知识储备欠缺的问题，我们建议学生阅读大量的参考书目及相关文献。通过阅读，学生对样品前处理、分离富集领域的理论知识和实验背景，由最初的了解到深入理解。鼓励学生参加课题组组会，由听学长讲文献及实验进展到自己做汇报，由相对被动到积极主动地融入课题组的科研讨论中。学生专业知识的广度和深度都有大幅度提高。

针对学生的实验技能欠缺的问题，我们通过指导学生动手开展实验来解决。通过合成样品前处理EP@PDA-TGDha COF材料，使学生掌握合成过程的实验技能，深入理解合成原理；通过对材料性能的表征，如：红外分析、核磁分析、粉末X射线衍射分析、扫描电子显微镜分析、热重分析、BET分析、Zeta电位分析、水接触角分析等，使学生掌握各种表征手段的原理、特性及仪器操作技能；通过样品前处理吸附去除实验，使学生掌握样品前处理实验技能，进一步探讨吸附机理，深入理解样品前处理过程的必要性；通过对实际样品测定，培养学生灵活运用理论知识、将理论转化为实际应用的能力。通过以上实验的开展，学生的实验技能得到了大幅提高。

通过“我爱实验室”科研训练，学生能够完成预先设定的实验目标，实验现象明显，具有较好的吸附应用效果。学生在实验过程中体验到科研探究的乐趣，科研思维能力有了明显的提高。同时，通过科研训练，学生切身体会到了“绿水青山就是金山银山”“保护环境，人人有责”。

## 4 结语

综上所述，本文开发了一种阳离子型共价有机框架气凝胶用于有机染料去除，并将该实验开发为“我爱实验室”科研训练实验。在教师的引导下，学生通过对实验背景知识的调研、材料合成和表征、样品前处理吸附过程的优化及应用于实际样品分析等实验，深入理解理论知识，掌握基本实验技能，探讨实验机理，并将理论转化为实际应用，切实践行了“学以致用，用以促学，学用相长”的教学理念。
